# Therapeutic Potential of Pterostilbene and Resveratrol on Biomechanic, Biochemical, and Histological Parameters in Streptozotocin-Induced Diabetic Rats

**DOI:** 10.1155/2018/9012352

**Published:** 2018-05-16

**Authors:** Bora Tastekin, Aykut Pelit, Sait Polat, Abdullah Tuli, Leman Sencar, Mustafa Muhlis Alparslan, Yusuf Kenan Daglioglu

**Affiliations:** ^1^Department of Biophysics, Faculty of Medicine, Çukurova University, Adana, Turkey; ^2^Department of Histology, Faculty of Medicine, Çukurova University, Adana, Turkey; ^3^Department of Biochemistry, Faculty of Medicine, Çukurova University, Adana, Turkey; ^4^Research and Practice Center of Experimental Medicine, Çukurova University, Adana, Turkey

## Abstract

**Aims:**

The aim of this study was to investigate the effects of pterostilbene (PTS) (trans-3,5-dimethoxy-4′-hydroxystilbene) and resveratrol (RSV) (trans-3,5,4′ trihydroxystilbene) applied at different doses for the treatment of streptozotocin- (STZ-) induced diabetic rats.

**Materials and Methods:**

At the end of the 5-week experimental period, the right gastrocnemius muscles of the rats were examined biomechanically, while the left ones were examined histologically. In addition, blood glucose, serum insulin, and malondialdehyde (MDA) levels were analyzed in blood samples taken from the rats.

**Results:**

The skeletal muscle isometric contraction forces, which showed a decrease with diabetes, were observed to increase with antioxidant applications. Blood glucose, serum insulin, and MDA levels in diabetic rats approached normal levels after applying PTS. When the electron microscopic images of the rat skeletal muscle were examined, those in the combination treatment group were observed to show a better enhancement in the skeletal muscle morphological structure compared to the other diabetic and treatment groups.

**Conclusion:**

According to the findings, we suggest that these antioxidant treatments might have good therapeutic nutraceutical potential for some muscle diseases that coexist with diabetes. These treatments should be comprehensively investigated in the future.

## 1. Introduction

Diabetes mellitus causes biomechanical and bioelectrical changes due to structural degeneration in skeletal muscles. It is still unclear what kind of degradation at the cellular level causes these changes. Muscular atrophy develops in skeletal muscles of diabetic people, which results in a decrease in their muscle efficiency [1, 2]. Diabetic myopathy is expressed as an unprotected state of muscle mass and is commonly seen in Type 1 DM and Type 2 DM patients [1, 3]. Studies have shown that the contraction and morphology of the skeletal muscles are irreversibly deteriorated by glucose and insulin imbalance in Type 1 DM [4, 5].

The antioxidant properties of RSV have been reported to be an important contributor to the removal of free radicals [6, 7]. PTS, on the other hand, has been proven to significantly reduce the pathological changes observed in the liver and kidney as a consequence of diabetes. The greater bioavailability of PTS (80%) compared to RSV (20%) makes the former potentially more advantageous as a therapeutic agent [8, 9]. Nutraceuticals used in medical applications are thought to have little or no side effects on metabolism and have received considerable attention in recent years due to their reliable utility in many pathological conditions, including diabetes [10, 11].

Both PTS and RSV treatments have a therapeutic effect against complications developing with diabetes. Common issues associated with these treatments are based on the analysis of enzyme activity, gene expression levels, protein quantities, and pancreatic immunohistochemical studies. A comprehensive review of related literature revealed no scientific studies showing the effects of antioxidants such as PTS and RSV on the contraction parameters and morphology of gastrocnemius muscle. Therefore, in this study, we aimed to investigate the biomechanical, biochemical, and histological properties of STZ-induced diabetic rats and to observe the treatment effects of PTS, RSV, and their combination (Mix) at different doses.

## 2. Materials and Methods

The care and use of animals and the experimental protocols of this study were approved by the Institutional Animal Care and Use Committee of Çukurova University.

### 2.1. Animals

Eighty male Wistar Albino rats weighing 250–300 g were used in this study. The animals were fed with standard rat pellet feed (ad libitum) and tap water. The illumination of the room where the animals were located was set for a 12/12 hour light-dark cycle. All animals were kept in this room at 22 ± 2°C and 40–60% humidity. After diabetes was successfully induced using STZ, animals with weak body resistance, excessive weight loss, and blood glucose above 500 mg/dl were excluded from the experiment due to diabetic complications to maintain standardization.

**Figure 1:**
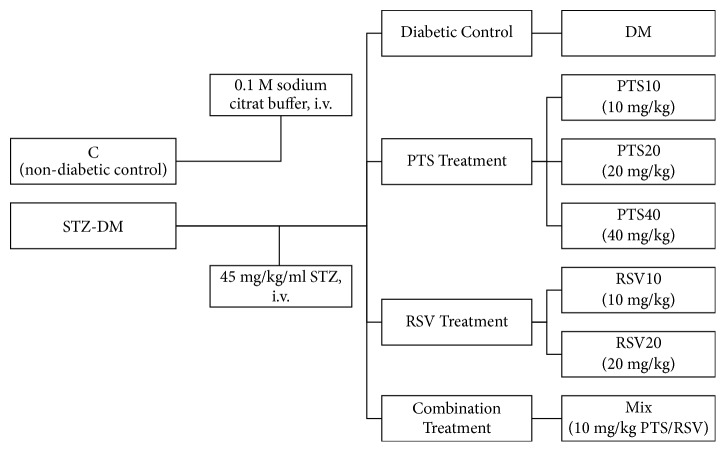
Schematic presentation of the experimental groups.

The rats were divided into 8 groups (in each group, *n* = 10), and the groups were defined as follows:

  C; non-diabetic rats injected (i.v.) with 0.1 M cold citrate buffer (pH 4.5),
  DM; single dose 45 mg/kg/ml STZ-induced diabetic rats (i.v.),
  PTS10; diabetic rats treated with 10 mg/kg body weight per day PTS (i.p.),
  PTS20; diabetic rats treated with 20 mg/kg body weight per day PTS (i.p.),
  PTS40; diabetic rats treated with 40 mg/kg body weight per day PTS (i.p.),
  RSV10, diabetic rats treated with 10 mg/kg body weight per day RSV (i.p.),
  RSV20; diabetic rats treated with 20 mg/kg body weight per day RSV (i.p.),
  Mix; diabetic rats treated with 10 mg/kg body weight per day combination of PTS and RSV (i.p.) (Figure 1).

PTS and RSV antioxidants were dissolved freshly in 10% dimethyl sulfoxide (DMSO) and administered intraperitoneally to the rats.

### 2.2. Experimental Induction of Diabetes

Diabetes in rats was induced by injection of a single dose of 45 mg/kg/ml STZ (Sigma Chemicals, St. Louis, MO, USA) dissolved in 0.1 M cold citrate buffer (pH 4.5) into the tail vein (i.v.). After 72 hours following the injection of STZ, diabetes was determined by measuring the blood glucose level from the tail using a glucometer (Accu-Chek Performa Nano, Roche Diagnostics, Mannheim, Germany). Rats with blood glucose level ≥ 300 mg/dL (16.7 mmol/l) were considered diabetic, and those with blood glucose below this value were not included in the study [12].

### 2.3. Treatment with PTS and RSV

The PTS and RSV antioxidants used in this study were obtained from Sabinsa (New Jersey, USA) as a research gift [13, 14]. The purity of each agent was >99%, as determined by HPLC. PTS and RSV were dissolved in 10% DMSO at amounts ≤ 30 mg/ml and ≤50 mg/ml, respectively, and i.p. injection was performed for all groups.

### 2.4. Solutions

Skeletal muscle preparations were stored in Krebs solution, similar to extracellular fluid. Biomechanical recordings were taken in this solution gassed with 95% O_2_ and 5% CO_2_ in an organ bath. One L of Krebs solution was prepared using the quantities of 118 mM NaCl, 4.69 mM KCl, 0.6 mM MgSO_4_, 1.17 mM KH_2_PO_4_, 11.1 mM Glucose, 25.0 mM NaHCO_3_, and 2.5 mM CaCl_2_. The temperature was fixed at 37°C, and the pH was ~7.4 [15].

### 2.5. Tissue Preparations

At the end of the 5-week experimental period, i.p. anesthesia was administered to the animals with 75 mg/kg sodium pentobarbital (Ulagay Pharmaceutical Industry Turkish Incorporated Company, Istanbul, TURKEY); their rib cages were opened, and the animals were sacrificed by draining blood from their hearts [16]. Of the quickly dissected gastrocnemius muscles, the right ones were used for biomechanical analysis, and the left ones were used for histological recordings. The right-leg gastrocnemius muscles were placed into the organ bath with the distal (bottom) and proximal (top, towards the transducer) ends of the muscle.

### 2.6. Biomechanical Measurements

The optimal length of each muscle placed vertically in the organ bath was determined after a 30-minute thermoregulation and equilibration period (the length gives the maximum muscle tension). The muscles were then placed between two electrodes made of platinum and stimulated simultaneously. The muscles were stimulated supramaximally for a duration of 0.5 ms with square frequencies of 0.05 Hz (15–20 V) to record their single twitch isometric contraction values. Later, maximum contraction responses were recorded with 0.5 ms pulses at frequencies of 10, 20, 50, and 100 Hz (15–20 V) after resting ~15–20 minutes before each stimulus [17]. To stimulate the muscle and record the response, a force–displacement transducer (FDT 10-A 500 g, Commat, Ankara, Turkey), stimulator (STPT02-A, Commat, Ankara, Turkey), research tissue organ bath and circulator (WBC 3044, Commat, Ankara, Turkey), and Biopac Systems MP30 (Goleta, CA, USA) were used. Specific muscle isometric contraction force (*sP*_*t*_, mN/mm^2^), contraction time (CT), half-relaxation time (HRT), contraction and relaxation rates (±*dP*/*dt*), and specific tetanic contraction force (*sP*_*o*_) were defined as the contraction parameters (Table 1). Muscle cross-sectional area was estimated from the muscle weight and length. Length was measured before the distal tendons were cut [18].

### 2.7. Biochemical Measurements

Serum insulin levels were determined with an enzyme-linked immunosorbent assay (ELISA) kit using rat insulin as the standard (Hangzhou Eastbiopharm Co. Ltd., Hangzhou, China). A pink colored mixture is formed as a result of the incubation of malondialdehyde and occurs as a secondary product after lipid peroxidation with thiobarbituric acid (TBA) at pH 3.4 and 95°C. The absorbance of this mixture, measured via spectrophotometer at 532 nm, gives a measurement of MDA levels. The determination of MDA values was carried out according to the method previously described by Yuksel et al. [19].

### 2.8. Electron Microscope Tracking Method

Gastrocnemius preparations taken for electron microscopic observations underwent a sophisticated scrutiny by the Department of Histology, Faculty of Medicine, Çukurova University. Sections that were 500 A° thick were taken from the blocks using a Reichert Ultracut S ultramicrotome and placed in 200- to 300-mesh copper grids. The sections were stained with saturated uranyl acetate in 70% ethyl alcohol and Reynolds lead citrate solutions. The stained sections were examined with a Transmission Electron Microscope (JEOL-JEM 1400, Japan) [20].

### 2.9. Statistical Analysis

Data obtained for the groups was expressed as the mean ± SEM. For each group, the number of animals included in the study is *n* = 10. The Kolmogorov-Simirnov test was administered for normality. One-way analysis of variance (ANOVA) was used to determine whether there are any statistically significant differences among the groups, and *p* < 0.05 was accepted value for statistical significance. The IBM SPSS Statistics version 20.0 software program was used (SPSS Inc., Chicago, IL, USA licensed by Çukurova University).

## 3. Results

### 3.1. Body Weight and Blood Glucose Levels

The weight loss of the animals differed significantly for the PTS, RSV, and non-diabetic control groups compared to the DM group (*p* < 0.05). There was no significant difference between the groups treated with PTS and RSV at the same dose of 10 or 20 mg/kg. (*p* > 0.05).

**Figure 2:**
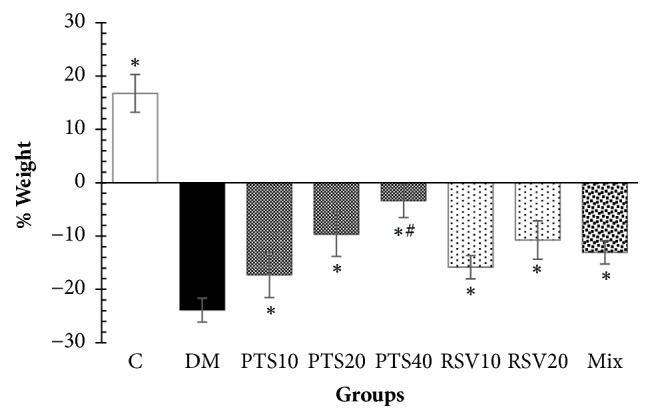
Percent of weight change from day 3 after STZ application to the end of 5th week, groups were compared within themselves. The results are given as percentage mean weight ± SEM. ^*∗*^Significantly different from DM (*p* < 0.05), ^#^significantly different from all other groups (*p* < 0.05).

**Figure 3:**
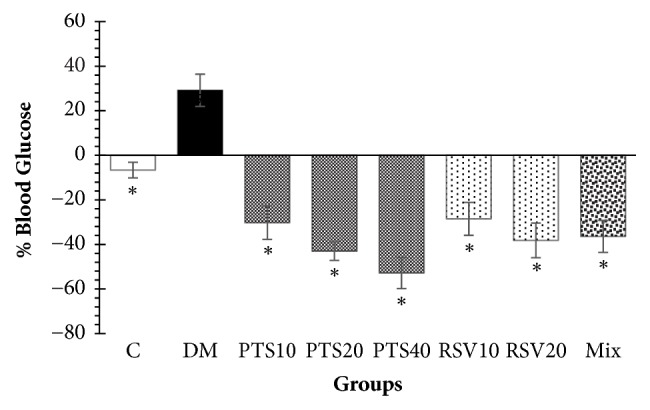
Percent change in blood glucose from day 3 after STZ application to the end of 5th week, groups were compared within themselves. The results are given as percentage mean blood glucose ± SEM. ^*∗*^Significantly different from DM (*p* < 0.05).

In addition, the weight changes of the animals in the PTS10, RSV10 and RSV20 groups did not show any significant differences when compared to the Mix group (*p* > 0.05), whereas those of the PTS20 and PTS40 groups were significantly different from that of the Mix group (*p* < 0.05) (Figure 2). On the other hand, the treatment with 40 mg/kg per day PTS (−52.8%) was observed to be the most effective treatment for the reduction of blood glucose levels (*p* < 0.05). There was no significant difference in blood glucose levels between the PTS10 and RSV10 (*p* = 1.00), PTS20 and RSV20 (*p* = 0.738), or PTS20 and PTS40 (*p* = 0.147) groups (Figure 3).

### 3.2. Isometric Twitch Contraction and Parameters

The isometric twitch contraction of group PTS40 was observed to be increased +67.44% compared to that of group DM and was increased +11.60% compared to C. In addition, when groups with the same dosage were examined, the isometric twitch contraction of the PTS10 and RSV10 groups, compared to the DM group, increased +21.1% and +12.2%, respectively. In the same way, the isometric twitch contraction of PTS20 increased +42.3% and RSV20 increased +27.6%, compared to DM. In addition, the PTS20 group showed +11.5% more isometric twitch contraction improvement than the RSV20 group while the PTS10 group showed +8.93% more isometric twitch contraction improvement than the RSV10 group. On the other hand, all of the diabetic groups treated with antioxidant treatment were significantly different from the DM group in terms of isometric twitch contraction (*p* < 0.05).

**Figure 4:**
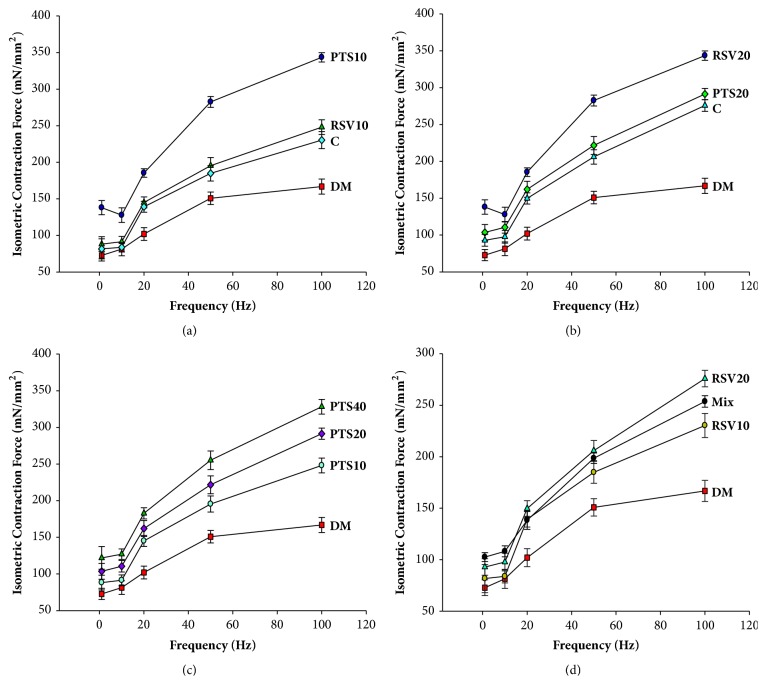
The responses of isometric contraction forces at the frequencies of 1, 10, 20, 50, and 100 Hz. The change in different antioxidants (a-b) administered at the same dose compared to C and DM, the changes among groups receiving 10, 20, and 40 mg/kg PTS (c) and 10, 20 mg/kg RSV and Mix (d) compared to DM. The results are given as the mean ± SEM.

The mean values of CT, which is one of the isometric contraction parameters, for the PTS10 (*p* = 0.06) and RSV10 (*p* = 0.164) groups were not significantly different from that of the DM group, whereas the mean values of CT for the other treatment groups were significantly different from that of the DM group (*p* < 0.05). For the HRT, we observed that the duration of the DM group was 22.0% more than that of C group. The reductions in HRT values, compared to DM, were concatenated: PTS40 (17.4%) > PTS20 (13.4%) > RSV20 (11.1%) > RSV10 (8.3%) > Mix (10.3%) > PTS10 (7.1%) (Table 1). The comparative values of the maximal contraction forces obtained from the groups after performing at 10, 20, 50, and 100 Hz frequencies are shown in Figure 4.

### 3.3. Serum Insulin and Serum MDA Levels

**Figure 5:**
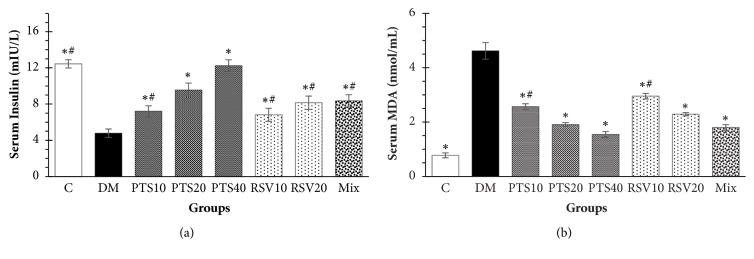
(a) Serum insulin (mIU/L) and (b) serum MDA values (nmol/mL). The results are given as the mean ± SEM. ^*∗*^Significantly different from group DM (*p* < 0.05), ^#^significantly different from PTS40 (*p* < 0.05).

The mean insulin level of DM decreased by 62.0% compared to C. Insulin levels of the diabetic rats in all treatment groups were observed to be significantly different from those in DM (*p* < 0.05). When the effects of doses administered at the same amount were examined compared to the diabetic group (non-treatment), it was observed that PTS10 compared to RSV10 (+5.94%) and PTS20 compared to RSV20 (+17.28%) increased the insulin improvement (Figure 5(a)). However, the levels of MDA in all groups except C increased depending on the increase of superoxide radicals in the formation of oxidative stress damaged diabetes. The administration of 40 mg/kg PTS in diabetic rats was more effective than the other treatment groups in scavenging the free radicals (Figure 5(b)). The percentage differences in MDA levels of the other groups compared to that of the DM group were PTS40 (−66.6%) > Mix (−61.0%) > PTS20 (−58.6%) > RSV20 (−50.4%) > PTS10 (−44.3%) > RSV10 (−36.1%).

### 3.4. Histological Examinations of PTS and RSV Treatments

**Figure 6:**
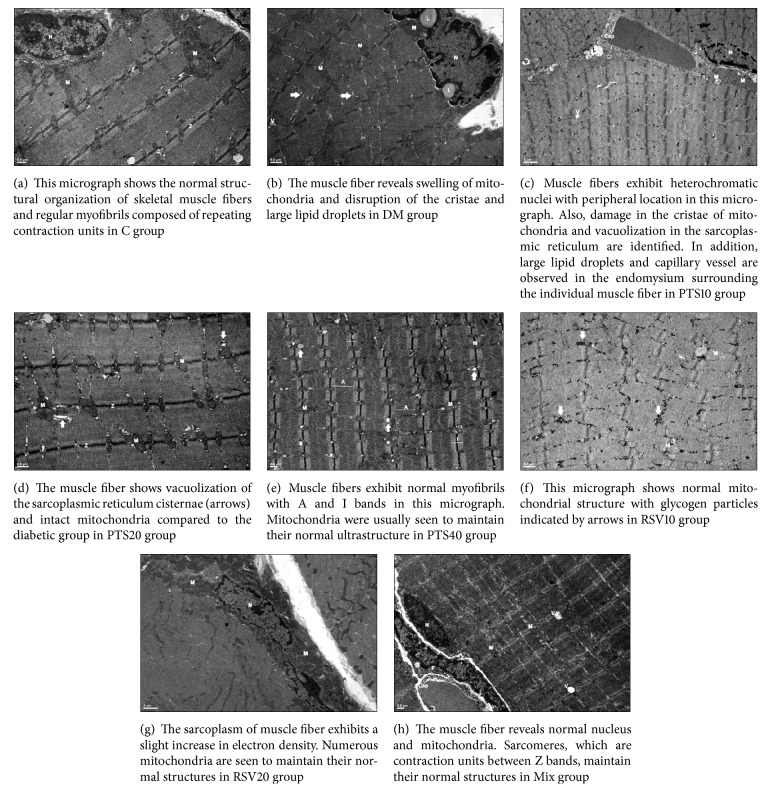
Electron microscopic images of the skeletal muscle tissue samples obtained from the groups. The groups C, DM, PTS10, PTS20, PTS40, RSV10, RSV20, and Mix have been enumerated from (a) to (h), respectively. M: mitochondria, N: nucleus, V: vacuolization, L: lipid droplets, Cap: capillary vessel, F: fibroblast, E: endomysium, Arrows in (b) and (d): sarcoplasmic reticulum cisternae, Arrows in (e) and (f): glycogen particles, Z: Z line, A and I: A and I bands, Bars in (a)–(d), (f), and (h): 0.5 *μ*m, and Bars in (e) and (g): 1 *μ*m.

Electron microscopic evaluation of the muscle tissues of the groups revealed that the cell nuclei had normal chromatin regulation in C. In addition, it was found that the chromatin in the cell nucleus existed intensely on the inner surface of the nucleus sheath, the nucleus had one or two nuclei, and the sarcomeres were in normal structure (Figure 6(a)). In DM, the presence of degenerative changes in the nucleus and organelles of muscle cells was observed and numerous lipid droplets with large diameters were detected close to the mitochondria. Furthermore, there was a pronounced vacuolization in the muscle cells of this group (Figure 6(b)). On the other hand, it was noted that all three groups (PTS10, PTS20, and PTS40) treated with PTS showed fewer degenerative changes resulting from defects in the nucleus and organelles of the skeletal muscle fibers and deterioration in myofibril regulation than the DM group. Glycogen and lipid droplets in the sarcoplasm were also observed to be significantly reduced in PTS treatment groups when compared with that of the DM group (Figures 6(c)–6(e)). In addition, degenerative changes in muscle fibers were found to be relatively reduced, and in particular, the myofibril organization was more regular in the RSV treatment groups compared with both the DM and PTS treatment groups (Figures 6(f)-6(g)). In the Mix group, although substantial changes in the crystals of some mitochondria and slight enlargement of the sarcoplasmic reticulum were observed in some cells, these organelles were found to have normal ultrastructural structures in many areas. The lipid droplets and cytoplasmic vacuolization in the muscle tissues of this group were prominently reduced compared to the diabetic and other treatment groups (Figure 6(h)).

## 4. Discussion

In this study, a biomechanical, biochemical, and histological examination of the effects of PTS and RSV within nutraceutical drugs, which have recently been used in many metabolic diseases, especially diabetes, were performed. Patients and practitioners prefer to use oral antidiabetic drugs for the prevention and treatment of the disease since it is difficult to use injectable insulin preparations. New nutraceuticals have increasingly intrigued researchers [21, 22]. Among these nutraceuticals, PTS and RSV are especially noteworthy because of their reduced side effects.

RSV antioxidant treatments are effective at increasing insulin sensitivity, developing mitochondrial functions, and providing protection against cardiovascular and neurodegenerative diseases. PTS is thought to have numerous protective and therapeutic properties in a wide range of human diseases, including neurological, cardiovascular, metabolic, and hematological disorders [23]. It is known that weight loss and blood sugar increase significantly in STZ-induced Type 1 diabetic rats [24, 25]. In our study, the body weight of the untreated diabetic group was reduced significantly. This reduction of body weight was also seen in PTS and RSV treated diabetic rats compared to the weight of the normal rats in control groups. Improved glycemic levels can be a consequence of higher glucose utilization by cells, which oxidize glucose as a metabolic energy source, thereby protecting adipose and muscle tissue and recovering body weight in diabetic animals treated with antioxidants. When the experimental groups were examined biomechanically and biochemically, it was seen that the endpoints of the group treated with 40 mg/kg PTS were closer to the normal values than those of the other groups were. Additionally, the combination therapy group administered at a dose of 10 mg/kg of each of PTS and RSV was found to be more effective, as examined histologically, than the other groups. In addition, while the administration of 20 mg/kg PTS was more effective than the same amount of RSV, 10 mg/kg RSV, a lower dose, was more effective than PTS for all parameters measured at the end of this experiment. The biomechanical and biochemical effects of the 10 mg/kg PTS/RSV combination therapy were observed to be almost similar to the corresponding effects of the 20 mg/kg dose of RSV treatment. Manickam et al. (1997) found that a 20 mg/kg dose of pterostilbene, isolated from Pterocarpus marsupium (PM), reduced body weight loss by 20% in STZ-induced rats [26]. This finding is consistent with our result that 20 mg/kg PTS reduced weight loss by 21.05% in STZ-induced diabetic rats. Our results are also consistent with the results of Satheesh and Pari, (2006) who found a 56.5% reduction in plasma glucose levels with the oral application of 40 mg/kg PTS for 6 weeks [27]. We found a 52.8% reduction in with the same dose of PTS. PM containing PTS has been particularly reported in some studies to enable the re-granulation of pancreatic beta cells [28, 29]. This finding suggests that beta cells destroyed in diabetic conditions may be renewed, and thus, PTS may contribute to this mechanism of anti-diabetic action.

In our study, we observed that serum insulin levels of diabetic rats without any treatment decreased by 62.0% compared to the control group. We assumed that this a natural consequence of the reduction in insulin release by the destruction of the pancreatic beta cells in Type 1 DM. It was also observed that PTS antioxidants provided a greater insulin increase than RSV at the same dosages, when we compared the effects of PTS and RSV antioxidants on serum insulin levels in STZ-induced experimental diabetic rats. Furthermore, 40 mg/kg PTS administration increased the insulin levels more than all other treatment groups and the C group (*p* < 0.05). We determined that the changes in serum insulin levels, based on the antioxidant dose applications, were similar to those of related studies [30–33]. It has been reported that free radicals cause metabolic damage and destructive effects on lipid structures in oxidative stress-induced diabetic rats [34]. In the present study, we observed that the MDA levels of the groups were decreased more by PTS than other treatments in the same number of applications. On the other hand, the combination antioxidant treatment was more effective than PTS and RSV applied doses of 10 and 20 mg/kg and showed a similar effect to administration of 40 mg/kg PTS.

The imbalances in blood glucose and insulin levels that occur with Type 1 DM can cause irreversible damage to the contractility and morphology of skeletal muscles [35]. In our study, the isometric contraction parameters of the gastrocnemius muscle in rats decreased significantly in the non-treated groups, but significant improvement was observed in the treated groups, especially with the treatment of 40 mg/kg PTS and 10 mg/kg PTS/RSV compared to the diabetic group. In addition, the CT and HRT of the rats in group DM were found to be prolonged compared to group C (*p* < 0.05). On the other hand, the maximal tetanic force values obtained from skeletal muscles for the groups treated with antioxidant treatment increased significantly (*p* < 0.05) in comparison with group DM and group C. We observed that tetanic contraction forces improvement was 3-4 times greater than the isometric contraction force, which correlates with the results from our previous study [17, 36].

In addition, skeletal muscle tissue samples of STZ-induced diabetic rats exhibited nuclear shrinkage, pyknosis, crystals of eroded mitochondria, degranulation, and numerous vacuoles. The distortions in the tissues suggest that insulin release and synthesis are inhibited [37, 38]. In the examination of electron microscopic images of the gastrocnemius muscle tissues of the rats in the experimental groups, we observed in our study that the degenerative changes observed in the DM group were ameliorated by antioxidant treatment. In the PTS treatment groups, the amount of connective tissues decreased in the perimysium surrounding the fascicles compared to the diabetic group. It was also noteworthy that inflammatory cells were reduced in this group compared to the diabetic group. In the RSV treatment groups, the perimysium surrounding the fascicles was found to be of relatively normal thickness. The fact remains that the presence of vacuolization in the sarcoplasm was detected in both PTS and RSV groups. In the RSV treatment groups, the localization of myofibril was more regular than that in the PTS and DM groups. Lipid droplets and cytoplasmic vacuolization in the combination therapy group of 10 mg/kg PTS/RSV were less than those in the DM and other treatment groups. Some researchers have assumed that resveratrol activated SIRT1-dependent signaling cascades to inhibit TGF-*β*1 signaling and decrease the acetyl histone H3 level in the muscle of mice. In addition, previous studies suggested that targeting SIRT1 as a key regulator of energy might improve the muscle pathology in patients with muscular dystrophies [39]. On the other hand, pterostilbene is known to have therapeutic potential through Nrf2 against pancreatic beta-cell apoptosis [9, 40]. It is thought that Nrf2 affects skeletal muscle morphology in a similar pathway as resveratrol.

## 5. Conclusion

Studies suggest that RSV has antioxidant effects at low doses and pro-oxidant effects at high doses [41]. For that reason, we used low doses (10, 20 mg/kg) of PTS and RSV to observe comparative effects in our study. Furthermore, we observed high doses (40 mg/kg) of PTS, which has rarely been studied and has been used as a treatment method more recently than resveratrol. In addition, we also evaluated the effects of low dose (10 mg/kg) PTS and RSV combination applications.

According to our findings, PTS and RSV treatments significantly improved the metabolic parameters of STZ-induced diabetes. We also found that the skeletal isometric contraction parameters, which showed a decrease with diabetes, were increased more by PTS administration than RSV. In addition, blood glucose, serum insulin, and MDA levels in diabetic rats were determined to be closer to normal with PTS application. In the examination of the skeletal muscle electron microscopic images of diabetic rats treated with antioxidant treatment, the healing effect of 10 mg/kg PTS/RSV combination therapy in Type 1 DM was observed to be comparatively better than that of the other groups. According to our experimental findings, PTS is relatively more effective than RSV at the same dose, which makes the former nutraceutically more advantageous.

## Figures and Tables

**Table 1:** Isometric contraction parameters. *sP*_*t*_: specific isometric contraction, CT: contraction Time, HRT: half relaxation time, +*dP*/*dt*: contraction rate in the positive direction, −dP/dt contraction rate in the negative direction, *sPo*: specific tetanic contraction force.

Groups	*sP*_*t*_, mN/mm^2^	CT, ms	HRT, ms	+*dP*/*dt*, mN/mm^2^·ms	−*dP*/*dt*, mN/mm^2^·ms	*sP*_*o*_, mN/mm^2^	*sP*_*t*_/*sP*_*o*_
C	138,0 ± 9,7^*∗*^	30,1 ± 0,5^*∗*^	28,6 ± 1,6^*∗*^	7461,4 ± 270^*∗*^	6575,9 ± 270^*∗*^	343,5 ± 6,4^*∗*^	0,40 ± 0,028
DM	72,8 ± 7,6^#^	42,4 ± 1,4^#^	34,9 ± 1,3^#^	4360,3 ± 291	3708,2 ± 302	166,8 ± 10,4^#^	0,46 ± 0,024
PTS10	88,2 ± 10,0^#^	39,7 ± 0,8^#^	32,4 ± 3,3	6270,0 ± 180^*∗*^	5398,8 ± 211^*∗*^	248,1 ± 10,2^*∗*#^	0,35 ± 0,032
PTS20	103,6 ± 10,7^*∗*#^	35,9 ± 0,5^*∗*#^	30,2 ± 1,2^*∗*^	6620,2 ± 310^*∗*^	5734,1 ± 236^*∗*^	291,4 ± 7,6^*∗*#^	0,35 ± 0,030
PTS40	121,9 ± 15,4^*∗*^	32,6 ± 2,4^*∗*^	28,8 ± 2,3^*∗*^	7084,9 ± 476^*∗*^	6279,7 ± 143^*∗*^	328,2 ± 10,1^*∗*^	0,37 ± 0,030
RSV10	81,7 ± 13,7^#^	37,3 ± 1,2^#^	32,0 ± 1,8	5842,4 ± 234^#^	5182,0 ± 164^*∗*^	230,4 ± 11,7^*∗*#^	0,35 ± 0,028
RSV20	92,9 ± 8,0^#^	36,9 ± 0,5^*∗*#^	31,0 ± 1,1^*∗*^	6315,7 ± 141^*∗*^	5580,0 ± 200^*∗*^	275,9 ± 8,1^*∗*#^	0,33 ± 0,025
Mix	102,5 ± 4,44^*∗*#^	36,1 ± 0,5^*∗*#^	31,3 ± 1,5	6421,4 ± 154^*∗*^	5559,4 ± 208^*∗*^	253,7 ± 5,6^*∗*#^	0,44 ± 0,027

The results are expressed as the mean ± SEM ^*∗*^significantly different from group DM (*p* < 0.05), ^#^significantly different from group C (*p* < 0.05).

## References

[1] Monaco C. M. F., Perry C. G. R., Hawke T. J. (2017). Diabetic Myopathy: Current molecular understanding of this novel neuromuscular disorder.

[2] López-Noriega L., Cobo-Vuilleumier N., Narbona-Pérez Á. J. (2017). Levothyroxine enhances glucose clearance and blunts the onset of experimental type 1 diabetes mellitus in mice.

[3] Hernández-Ochoa E. O., Llanos P., Lanner J. T. (2017). The Underlying Mechanisms of Diabetic Myopathy.

[4] Nutter C. A., Jaworski E., Verma S. K., Perez-Carrasco Y., Kuyumcu-Martinez M. N. (2017). Developmentally regulated alternative splicing is perturbed in type 1 diabetic skeletal muscle.

[5] Lima-Fontes M., Costa R., Rodrigues I., Soares R. (2017). Xanthohumol Restores Hepatic Glucolipid Metabolism Balance in Type 1 Diabetic Wistar Rats.

[6] Berman A. Y., Motechin R. A., Wiesenfeld M. Y., Holz M. K. (2017). The therapeutic potential of resveratrol: a review of clinical trials.

[7] Szkudelski T., Szkudelska K. (2015). Resveratrol and diabetes: from animal to human studies.

[8] Tsai H.-Y., Ho C.-T., Chen Y.-K. (2017). Biological actions and molecular effects of resveratrol, pterostilbene, and 3′-hydroxypterostilbene.

[9] Bhakkiyalakshmi E., Sireesh D., Sakthivadivel M., Sivasubramanian S., Gunasekaran P., Ramkumar K. M. (2016). Anti-hyperlipidemic and anti-peroxidative role of pterostilbene via Nrf2 signaling in experimental diabetes.

[10] Pandey M. M., Rastogi S., Rawat A. K. S. (2013). Indian traditional ayurvedic system of medicine and nutritional supplementation.

[11] Bacha U., Nasir M., Iqbal S., Anjum A. A. (2017). Nutraceutical, Anti-Inflammatory, and Immune Modulatory Effects of.

[12] Sheweita S. A., Mashaly S., Newairy A. A., Abdou H. M., Eweda S. M. (2016). Changes in oxidative stress and antioxidant enzyme activities in streptozotocin-induced diabetes mellitus in rats: Role of alhagi maurorum extracts.

[13] Cheng T.-C., Lai C.-S., Chung M.-C. (2014). Potent anti-cancer effect of 39-hydroxypterostilbene in human colon xenograft tumors.

[14] Yeo S. C. M., Ho P. C., Lin H.-S. (2013). Pharmacokinetics of pterostilbene in Sprague-Dawley rats: the impacts of aqueous solubility, fasting, dose escalation, and dosing route on bioavailability.

[15] Eshima H., Tamura Y., Kakehi S. (2017). Long-term, but not short-term high-fat diet induces fiber composition changes and impaired contractile force in mouse fast-twitch skeletal muscle.

[16] Fortes M. A. S., Pinheiro C. H. J., Guimarães-Ferreira L., Vitzel K. F., Vasconcelos D. A. A., Curi R. (2015). Overload-induced skeletal muscle hypertrophy is not impaired in STZ-diabetic rats.

[17] Pelit A., Emre M., Dağli K., Tuli A. (2013). The Impact of Magnesium on Isometric Twitch Parameters and Resting Membrane Potential of the Skeletal Muscle in Diabetic Rats.

[18] Cameron N. E., Cotter M. A., Robertson S. (1990). Changes in skeletal muscle contractile properties in streptozocin-induced diabetic rats and role of polyol pathway and hypoinsulinemia.

[19] Yuksel Y., Guven M., Kaymaz B. (2016). Effects of Aloe Vera on Spinal Cord Ischemia–Reperfusion Injury of Rats.

[20] Öz Gergin Ö., Yildiz K., Bayram A. (2015). Comparison of the myotoxic effects of levobupivacaine, bupivacaine, and ropivacaine: An electron microscopic study.

[21] Davi G., Santilli F., Patrono C. (2010). Nutraceuticals in diabetes and metabolic syndrome.

[22] Ozmen O., Topsakal S., Haligur M., Aydogan A., Dincoglu D. (2016). Effects of caffeine and lycopene in experimentally induced diabetes mellitus.

[23] Dvorakova M., Landa P. (2017). Anti-inflammatory activity of natural stilbenoids: A review.

[24] Roh S., Kwon O. J., Yang J. H. (2016). Allium hookeri root protects oxidative stress-induced inflammatory responses and *β*-cell damage in pancreas of streptozotocin-induced diabetic rats.

[25] Yorek M. S., Coppey L. J., Shevalye H., Obrosov A., Kardon R. H., Yorek M. A. (2016). Effect of Treatment with Salsalate, Menhaden Oil, Combination of Salsalate and Menhaden Oil, or Resolvin D1 of C57Bl/6J Type 1 Diabetic Mouse on Neuropathic Endpoints.

[26] Manickam M., Ramanathan M., Farboodniay Jahromi M. A., Chansouria J. P. N., Ray A. B. (1997). Antihyperglycemic activity of phenolics from Pterocarpus marsupium.

[27] Satheesh M. A., Pari L. (2006). The antioxidant role of pterostilbene in streptozotocin-nicotinamide- induced type 2 diabetes mellitus in Wistar rats.

[28] Rizvi S. I., Mishra N. (2013). Traditional Indian medicines used for the management of diabetes mellitus.

[29] Halagappa K., Girish H. N., Srinivasan B. P. (2010). The study of aqueous extract of Pterocarpus marsupium Roxb. on cytokine TNF- in type 2 diabetic rats.

[30] Elango B., Dornadula S., Paulmurugan R., Ramkumar K. M. (2016). Pterostilbene Ameliorates Streptozotocin-Induced Diabetes through Enhancing Antioxidant Signaling Pathways Mediated by Nrf2.

[31] El-Awdan S. A., Abdel Jaleel G. A., Saleh D. O. (2013). Grape seed extract attenuates hyperglycaemia-induced in rats by streptozotocin.

[32] Tamaddonfard E., Farshid A. A., Asri-Rezaee S. (2013). Crocin improved learning and memory impairments in streptozotocin-induced diabetic rats.

[33] Waly M., Guizani N., Suresh S., Rahman M. (2015). Ginger extract attenuates preliminary steps of streptozotocin-mediated oxidative stress in diabetic rats.

[34] Wojnar W., Kaczmarczyk-Sedlak I., Zych M. (2017). Diosmin ameliorates the effects of oxidative stress in lenses of streptozotocin-induced type 1 diabetic rats.

[35] Mukundwa A., Mukaratirwa S., Masola B. (2016). Effects of oleanolic acid on the insulin signaling pathway in skeletal muscle of streptozotocin-induced diabetic male Sprague-Dawley rats.

[36] Pelit A., Özaykan B., Tuli A., Demirkazik A., Emre M., Günay I. (2008). The effects of magnetic field on the biomechanics parameters of soleus and extensor digitorum longus muscles in rats with streptozotocin-induced diabetes.

[37] Arulselvan P., Subramanian S. P. (2007). Beneficial effects of *Murraya koenigii* leaves on antioxidant defense system and ultra structural changes of pancreatic *β*-cells in experimental diabetes in rats.

[38] Umrani R. D., Paknikar K. M. (2015). Jasada bhasma, a zinc-based ayurvedic preparation: Contemporary evidence of antidiabetic activity inspires development of a nanomedicine.

[39] Hori Y. S., Kuno A., Hosoda R. (2011). Resveratrol ameliorates muscular pathology in the dystrophic mdx mouse, a model for Duchenne muscular dystrophy.

[40] Bhakkiyalakshmi E., Shalini D., Sekar T. V., Rajaguru P., Paulmurugan R., Ramkumar K. M. (2014). Therapeutic potential of pterostilbene against pancreatic beta-cell apoptosis mediated through Nrf2.

[41] Kuršvietienė L., Stanevičienė I., Mongirdienė A., Bernatonienė J. (2016). Multiplicity of effects and health benefits of resveratrol.

